# Optimizing Care for High-Risk Multiple Pregnancy with POCUS – A Case of Quadruplet Pregnancy Early Diagnosis

**DOI:** 10.24908/pocus.v8i2.16562

**Published:** 2023-11-27

**Authors:** Bernardo Vidal Pimentel, Christopher Tsoutsoulas, Kristin Lythgoe, Frank Myslik

**Affiliations:** 1 Department of Internal Medicine, Hospital da Luz Lisboa Lisbon Portugal; 2 Division of Emergency Medicine, Department of Medicine, University of Toronto Toronto, Ontario Canada; 3 North York General Hospital Toronto, Ontario Canada; 4 Division of Emergency Medicine, Western University London, Ontario Canada; 5 London Health Sciences Centre London, Ontario Canada

**Keywords:** Point-of-Care Ultrasound (POCUS), Obstetrics and gynecology, Multiple pregnancy, Quadruplet pregnancy, In vitro fertilization

## Abstract

Managing multiple pregnancies is challenging and requires careful evaluation. Point of care ultrasound (POCUS) has emerged as a potentially crucial tool in assessing suspected first-trimester pregnancies. However, its role in evaluating multiple pregnancies remains uncertain. We present the case of a 36-year-old Ghanaian female who presented with acute vaginal bleeding after undergoing in vitro fertilization. A bedside transabdominal POCUS identified four intrauterine gestations with fetal poles and cardiac activity, suggesting a quadruplet viable pregnancy. A subsequent transvaginal ultrasound confirmed the findings. The patient was discharged with a follow-up appointment with an Obstetrician-Gynecologist. This case highlights the significance of POCUS in early pregnancy diagnosis, facilitating accurate identification and appropriate referral for further management. It also demonstrates the utility of POCUS in determining gestational age and viability. To our knowledge, no published case reports specifically address the diagnosis of a quadruplet pregnancy, emphasizing the role of POCUS in optimizing care for high-risk multiple pregnancies.

## Introduction

Point of care ultrasound (POCUS) has evolved into an invaluable tool for numerous healthcare professionals, spanning from the emergency department (ED) to the internal medicine ward 1. Obstetric POCUS has proven beneficial in the ED by offering real-time visualization and diagnostic information at the bedside 2. POCUS has significant enhanced obstetric care, particularly in resource-limited, remote, and austere environments 3, 4. While its utility in various obstetric conditions has been well-documented, there remains a scarcity of published articles specifically addressing the diagnosis and management of multiple gestations using POCUS. 

## Clinical Case

A 36-year-old Ghanaian female presented to our tertiary academic hospital's emergency department with a chief complaint of vaginal bleeding. She disclosed having undergone in vitro fertilization (IVF) one-month prior in Ghana due to a 6-year history of infertility. 

At triage, her vital signs were temperature of 36.9 °C, heart rate of 83 bpm, blood pressure of 122/84 mmHg, respiratory rate of 18 bpm and SpO2 of 99%. Physical examination revealed a non-tender abdomen and a closed cervical os with no active bleeding. 

**Figure 1:**
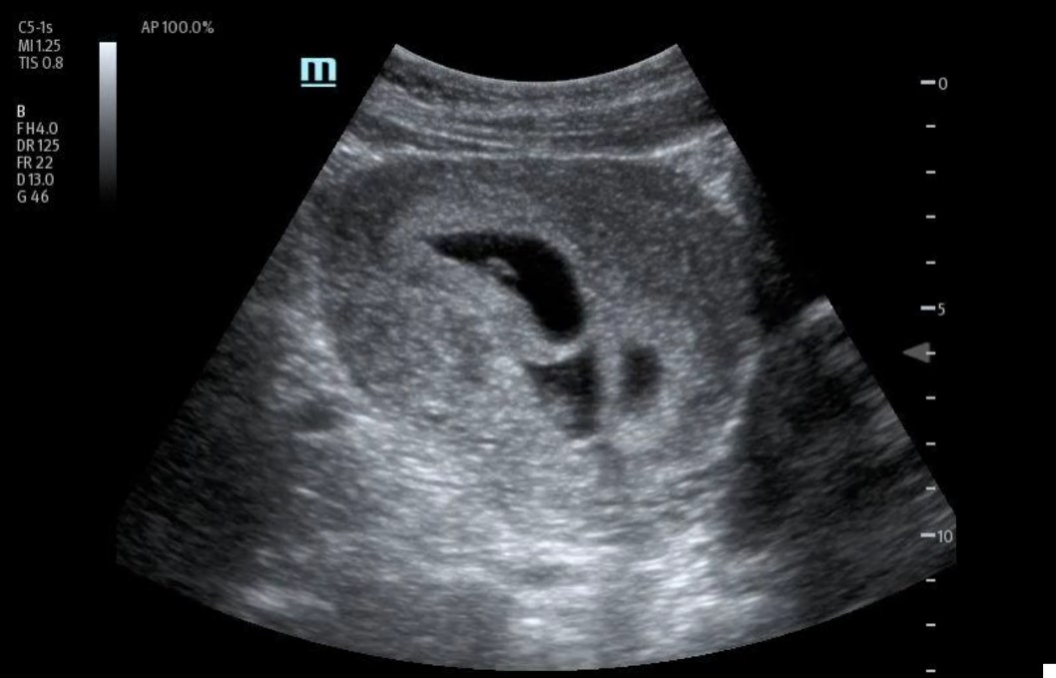
Bladder uterine juxtaposition.

**Figure 2:**
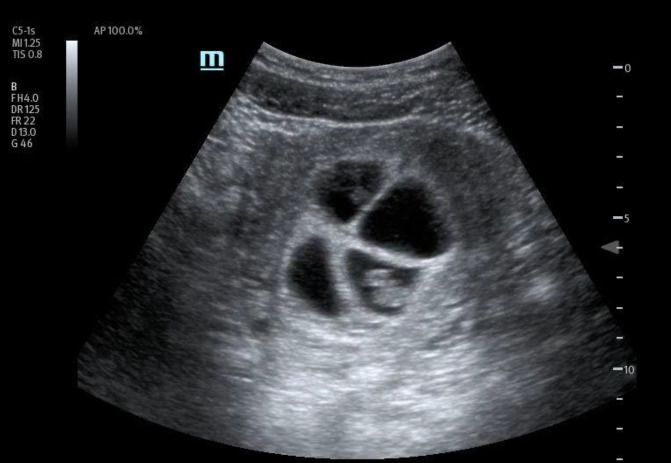
Four gestational sacs.

**Figure 3:**
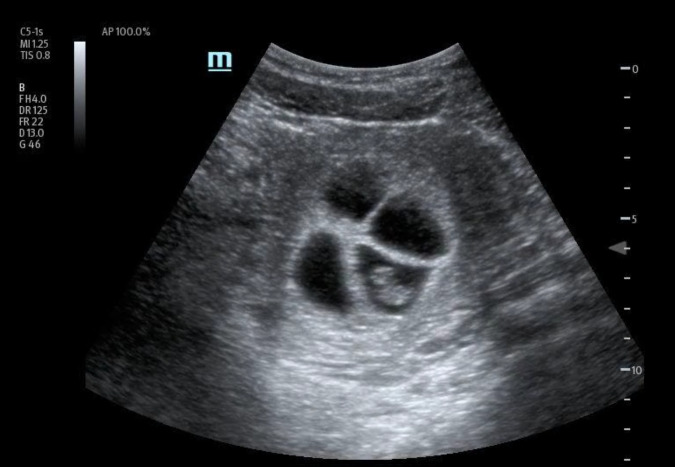
Left inferior fetal pole.

**Figure 4:**
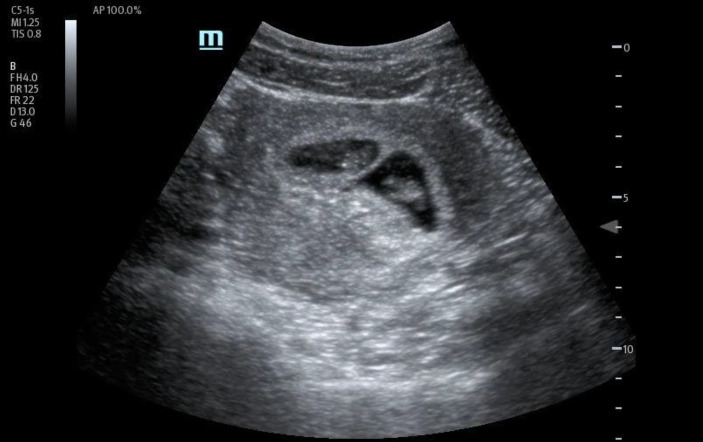
Right and left superior fetal poles.

**Figure 5:**
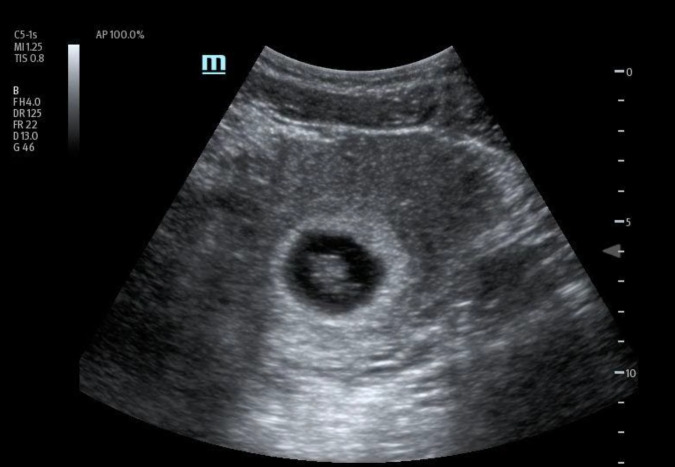
Right inferior fetal pole.

**Figure 6:**
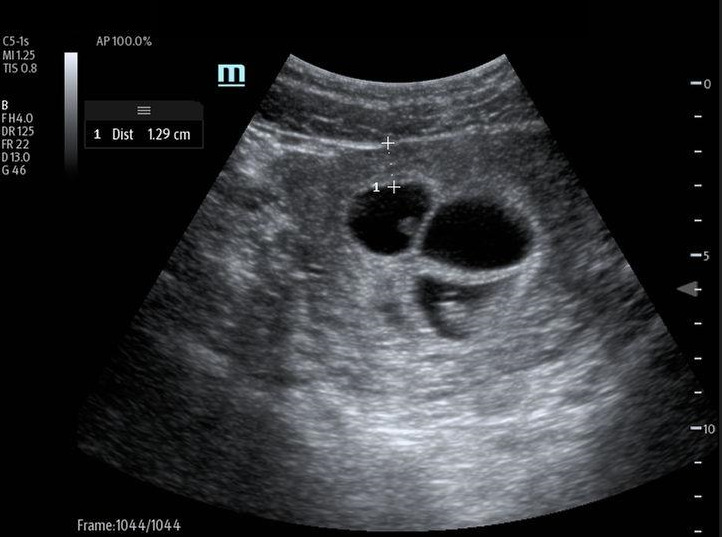
Myometrial Mantle Thickness.

In order to further evaluate the patient's condition, an obstetric POCUS examination was performed using a 3-5MHz curvilinear transducer. The POCUS examination identified the presence of four distinct live intrauterine gestations, each displaying a fetal pole and cardiac activity (Figures 1 to 6 and supplemental Video S1).

A serum beta human chorionic gonadotropin level subsequently drawn revealed a value of 377, 520 IU/L (<5 UI). A comprehensive obstetric ultrasound was further requested and confirmed the four viable intrauterine gestations, with estimated gestational ages ranging from 8 weeks 0 days to 8 weeks 2 days. There was no evidence of heterotopic ectopic pregnancy. 

Following the diagnosis, the patient was discharged from the emergency department with an urgent appointment booked with the on-call Obstetrician-Gynecologist for further management and follow-up. Due to the POCUS and comprehensive ultrasound exams identifying multiple gestations, we were able to provide the patient urgent follow up for her high-risk pregnancy. Typically, first trimester patients with vaginal bleeding at our center are referred to our Early Pregnancy Assessment Unit (EPAU), which does not always necessitate a call to the on-call Obstetrician-Gynecologist. 

Additionally, we hereby declare that the referred patient provided us with her verbal consent and had the opportunity to review this manuscript.

## Discussion

Multiple pregnancies including quadruplet pregnancies are rare and associated with increased risks of adverse maternal and neonatal outcomes 5. Multiple pregnancies following assisted reproductive technology are associated with similar if not higher risks such as ectopic or heterotopic pregnancy and ovarian hyperstimulation syndrome 6. The accurate identification of multiple gestations such as quadruplet pregnancies through POCUS enables healthcare providers to initiate timely and appropriate prenatal care, including close monitoring and management of potential complications 2, 3, 4.

Our case file highlights the utility of POCUS in providing crucial information regarding gestational age and viability in a patient presenting with first-trimester vaginal bleeding. Prior to our examination, the patient was unaware that she was pregnant. Furthermore, POCUS facilitated early diagnosis and referral for high-risk prenatal care. Early initiation of prenatal care has been associated with positive outcomes, including reduced neonatal and infant mortality rates and decreased incidence of low birth weight 7. 

It is important to acknowledge the limitations of transabdominal obstetric POCUS. Firstly, it should be noted that transabdominal POCUS cannot definitively rule out ectopic pregnancy. A recent systematic review encompassing both transabdominal and/or transvaginal POCUS examinations performed by emergency physicians reported a sensitivity of only 90% 8. Secondly, transabdominal ultrasound demonstrates lower diagnostic accuracy compared to transvaginal ultrasound, particularly in cases of ectopic tubal pregnancy 9, 10, 11. Third, it typically can only confirm the diagnosis of intrauterine gestation after the 6-8^th^ week of pregnancy, particularly when the fetal heart rate becomes detectable around the 8^th^ week 12. As a result, providers should not solely rely on POCUS as a substitute for comprehensive obstetric ultrasound examinations.

To our knowledge, this is the first case report to identify four live intrauterine gestations using obstetric POCUS. Utilizing POCUS in patients with high-risk multiple pregnancies who have undergone assisted reproduction can aid in the assessment and management of potential complications. 

In this case, POCUS correctly identified multiple viable live intrauterine gestations and helped facilitate appropriate prenatal care and follow-up. 

## Disclosures

The authors have no disclosures related to this work.

## Supplementary Material

 Video S1 Transabdominal ultrasound in the longitudinal axis, fanning from medial to lateral, with visualization of the 4 gestational sacs with the respective fetal poles.
